# When dreams don’t come true

**DOI:** 10.7554/eLife.79182

**Published:** 2022-04-13

**Authors:** Adna Dumitrescu

**Affiliations:** 1 Healthcare Improvement Scotland Edinburgh United Kingdom

**Keywords:** sparks of change, careers, identity, scientist

## Abstract

As she closes the door on her time in academia, a neuroscientist faces unexpected grief.

“So, what do you do for a living?”

I used to love answering that question. I’d get an anticipatory rush of endorphins as I would casually reply “I’m a scientist.” My answer would lure people to ask: “Oh, what type of science?”, and, with enough momentum built up, I would finally let it out: “I’m a neuroscientist actually. I study brains.” I enjoyed their look of surprise as they learned that this young-looking woman with an out-of-place accent was a researcher. If I’m being honest, I was still surprised I had managed to become one as well.

I originally wanted to be a therapist, but a month into the course I realised that my poor listening skills and impatient personality were ill-matched for that job. Instead, I switched to a degree in Cognitive Science. As I learned about positive and negative ions flowing across fatty membranes to create action potentials and charge us with electricity, I experienced something close to a religious revelation. I always believed that we are the sum of our biological parts – dualists be damned; this experience was when I narrowed it down to the idea that we *are* our brain. I became a determined neuro-preaching zealot and elbowed my way into a neuroscience PhD to see how I could record, control and play with action potentials. At that point, I made a pact with myself: I would give it my all and aim to have my own lab in ten years. Yet eight months short of this original deadline, during my second postdoc, I decided to leave academia.

**Figure 1:**
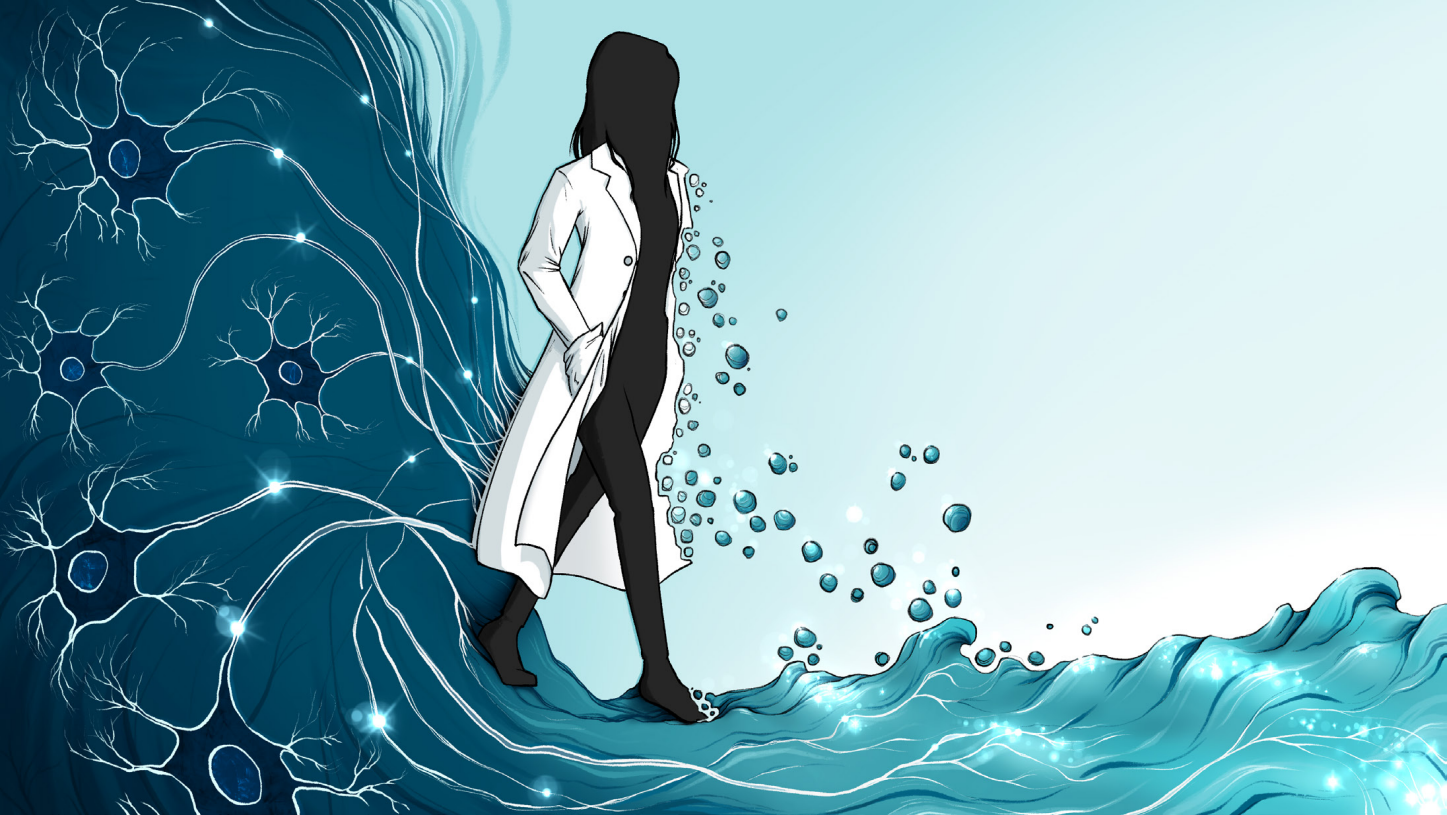
Giving up on her academic career forced a neuroscientist to put her brain ‘back into her body’ and to reflect on what it means to be a scientist.

Image credit: Marzia Munafo; @munafomarzia (CC BY 4.0).

For that entire decade, I became absorbed in my research, essentially working all the time. I did not complain about it then, and I will not start now. As a working-class Romanian immigrant, I felt like I had won the lottery. I had a rewarding job, which paid more than my parents ever made and which was, at times, exhilarating. I sang to myself for hours in dark microscope rooms, swore at cells which did not want to get patched, high-fived myself when they did. My favourite part was unblinding my experimental conditions, and finally figuring out if the effect direction would smash or confirm my hypothesis. It felt like being on a rollercoaster water ride, just exciting enough that you’re on the edge of screaming with excitement. I got to be the first person in the world to create this *one* specific piece of knowledge, no matter how small it was or that only twelve other people across the world cared about it as much as I did.

When I finally left academia, I did not expect to feel distraught. But there I was, 10 pm on a Thursday during the first year of our pandemic, wine glass in hand and cats doing annoying cat things in the background. I was watching a documentary about black holes with my partner, and on the screen, scientists gushed about how they couldn’t wait to find the results of their years-long project. The work had been hard, exhausting even, and they were almost at the finish line. They made it, and I burst into tears. I had recently handed in my resignation and for the first time, it hit me: never again will I feel the thrill of unblinding my experiments. This broke me. Sometimes dreams don’t come true. My dream went bust, and I got hurt in the process.

I started seeing a therapist to make sense of this pain. I knew that it was all in my brain, but why was my chest feeling heavier than usual, why was I sighing and crying at black holes? I was a brain floating in a metaphorical vat, which had tried to understand other brains for nearly a decade. Over an entire summer of gentle nudges, she helped me put it back into my body. She did the cliche ‘therapist thing’ and asked about my childhood; I wished she could have shocked my neurons into being less sad instead. She mentioned that I was grieving the loss of my career; I snickered that it was an over exaggeration – nobody had died!

Despite my initial resistance, it finally clicked one evening, during a rainy cycle home after my 8 pm session. I was in fact crying over losing parts of myself, the parts that loved to understand how things worked. Spy, fashion model, journalist, therapist: I still remember, in chronological order, all the professions I wanted to do as a child. Scientist was not on the list. Yet I was 10 years old when I did my first experiment, investigating whether God was real by lying to an Orthodox priest during confession and examining the consequences. I was 12 when I built my first logical, science-based argument using a month of slow internet research to convince my parents that it was safe — and even morally valid — for me to be a vegetarian. I was a scientist even before I had that title.

This realisation, in the end, took the sting out of my career change. My curiosity has been burning strong for 30 odd years; it will not peter out just because I don’t get paid to pipette small amounts of clear liquid into tubes, or to prod living neurons with electrodes. I might be a retired neuroscientist now, but I will never really stop wanting to understand how things work.

## Share your experiences

This article is a Sparks of Change column, where people around the world share moments that illustrate how research culture is or should be changing. Have an interesting story to tell? See what we’re looking for and the best ways to get in touch here.

